# Suppression of lung cancer in murine model: treated by combination of recombinant human endostsatin adenovirus with low-dose cisplatin

**DOI:** 10.1186/1756-9966-28-31

**Published:** 2009-03-05

**Authors:** Rui Z Bai, Yang Wu, Quan Liu, Ke Xie, Yu Q Wei, Yong S Wang, Kang Liu, Yan Luo, Jing M Su, Bing Hu, Ji Y Liu, Qiu Li, Ting Niu, Zhi W Zhao, Li Yang

**Affiliations:** 1 State Key Laboratory of Biotherapy and Cancer Center, West China Hospital, West China Medical School, Sichuan University, Keyuan Fourth Road, Chengdu, Sichuan, PR China; 2 Department of Oncology, Sichuan Academy of Medical Sciences, Sichuan Provincial People's Hospital, Chengdu, 610072, PR China

## Abstract

**Background:**

The sustained growth of tumors necessitates neovascularization. As one of the potent endogenous vascular inhibitors, endostatin has been widely used in antiangiogenesis therapy for tumor. Cisplatin is normally administered in chemotherapy for lung cancer but accompanied with serious side effects. In the current study, we investigated a novel chemo-antiangiogenesis therapeutic strategy to both improve toxic effects on lung cancer cells and reduce damages to normal cells in the anti-tumor therapy.

**Methods:**

In vitro, we transduced LLC cells with Ad-hEndo and collected supernatants. Western blotting analysis of the supernatants revealed expression of endostatin. In vivo, to fully investigate the suppression effect on murine lung cancer of the combination therapy, we injected recombinant human endostatin adenovirus intratumorally plus a low dose of cisplatin intraperitoneally routinely. The tumor volume and survival time were observed. Angiogenesis was apparently inhibited within the tumor tissues and on the alginate beads. Assessment of apoptotic cells by the TUNEL assay was conducted in the tumor tissues.

**Results:**

The combination treatment significantly suppressed the tumor growth and prolonged survival time of the murine LLC tumor model. This anti-tumor activity was associated with decreased microvessel density and increased apoptotic index of tumor cells.

**Conclusion:**

According to the results in this study, recombinant human endostatin adenovirus in combination with a low dose of cisplatin demonstrated apparent synergistic anti-tumor activity without marked toxicity. Thus, these observations may provide a rational alternative for lung cancer treatment.

## Background

The blood vessel formation plays an essential role in a large variety of physiological and pathological conditions. Numerous studies have shown that growth and progression of most solid cancers are ngiogenesis-dependent [1-4]. Neovascularization includes multiple complex sequential steps: degradation of basement membranes, proliferation and migration of endothelial cells, and deposition of basement membranes. Tumor angiogenesis is strongly regulated by both positive and negative factors in tumor growth, including a few growth factors such as VEGF, MMPs, and bFGF that regulate proliferation, migration and adhesion of endothelial cells. One of the potent endogenous angiogenesis inhibitors, endostatin, is a cleavage fragment containing COOH-terminal 184 amino acids of the basement membrane collagen XVIII. This product inhibits endothelial cell migration and proliferation, and then induces regression of tumors[5].

The theory of antiangiogenesis has been set forth by Folkman and others since the 1970s. It has advocated that suppressing tumor-related angiogenesis and thus depriving tumors of supply lines (of essential nutrients and oxygen) leads to a "dormant" state in which tumor cell proliferation and tumor expansion is stalled. In recent years, there have been quite a few published reports showing promising efficacy of endostatin protein in both cancer research and cancer clinical trials [6-8].

With the highest rates of morbidity and mortality among malignant tumors, lung cancer is one of the most common types of cancer threatening public health. Chemotherapy has been the leading treatment for cancer for a long time. And cisplatin is administered frequently in chemotherapy for lung cancer. However, the conventional chemotherapy is often accompanied by serious side effects, such as myelosuppression, kidney toxicity and nausea, leading to give-up of anti-tumor treatment. In the past decade, some other new cytotoxic drugs have come into clinic application. Despite the progress, chemotherapy has not satisfied expectation of complete responses to the therapy in patients or achieved cures in patients with advanced-stage cancer, which limited its application in clinical practice.

Besides traditional treatments such as chemotherapy, new cancer treatment strategies have been developed in recent years. An approach combining low-dose chemotherapy with antiangiogenesis factors has been reported to be potent in treatment of established animal tumors. Widely applied to inhibit cancer angiogenesis, gene therapy, especially adenovirus gene therapy shows no disadvantages of recombinant protein injection[9,10]. In the current research, we evaluated efficacy of combination of recombinant endostatin adenovirus with a low dose of cisplatin in treatment of murine Lewis lung cancer.

## Methods

### Construction of recombinant adenovirus

Construction of recombinant human endostatin adenovirus has been described in the previous study[8]. In brief, the endostatin cDNA encoding C-terminal 184 amino acids of human collagen XVIII was amplified by RT-PCR. After sequence confirmation, the cDNA was firstly cloned into the cloning vector PUC18 and then into a shuttle vector for rescue of recombinant adenovirus (using the AdEasy system). The recombinant adenovirus was constructed and purified in our lab.

### Cell Culture and viral preparation

Human embryonic kidney cell line (HEK293) and Lewis lung cancer cells (LLC) were obtained from the American Type Culture Collection (ATCC). They were cultured in DMEM supplemented with 10% fetal bovine serum (FCS) plus 1% amikacin routinely. The cultures were split 1:3 every 4 days. The viral particles were amplified in 293 cells, purified by CsCl gradient ultracentrifugation and measured by absorption (at A260). The virus titer was quantified using the standard TCID50 assay.

### Western Blotting of transfected cells supernatants in Vitro

LLC cells were transduced with Ad-hEndo and the control virus, Ad-null (both at MOI 100, 10^8^pfu per 10^6 ^cells in 1.0 ml complete medium) or involved no transduction. After the cells were conditioned at 37°C for 48 h, supernatants were harvested and concentrated by ultrafilter (centricon YM-3, Millipore), and were mixed with the same volume of 2× SDS (sodium dodecyl sulfate) sample buffer. Samples were separated on a 12% SDS-PAGE gel and transferred onto a PVDF membrane (polyvinylidene difluoride, BIO-RAD). After the cells were blocked by TTBS (0.1%Tween-20 in TBS) with 5% defatted milk for 1 h, the membrane was probed with rabbit antihuman endostatin serum (1:100) overnight at 4°C. Later the cells were incubated with 1:5000 horseradish peroxidase-conjugated anti-rabbit immunoglobulin (Sigma-Aldrich, St. Louis, MO, US). Protein bands were visualized using the DAB detection kit (Sigma-Aldrich, St. Louis, MO, US).

### Animal experiments

Female (6–8 weeks old) C57BL/6 mice (purchased from the Laboratory Animal Center of Sichuan University, Chengdu, Sichuan, China) were acclimated for one week and were fed with animal chow and water ad libitum. The mice were anesthetized prior to all procedures and observed until fully recovery.

The C57BL/6 mice of 6–8 weeks were injected s.c. with 1 × 10^6 ^LLC cells in 100 μl PBS in the right flank. 7 d later, when the tumors were palpable, the mice were randomly divided into 5 groups (n = 5 animals/group): Ad-hEndo, intratumoral injection of 1 × 10^9^pfu/100 μl recombinant adenovirus; cisplatin, intraperitoneal treatment of 1 mg/kg/100 μl; Ad-hEndo plus cisplatin, Ad-hEndo delivery locally, along with cisplatin administration intraperitoneally; empty virus, Ad-null, intratumoral injection of 1 × 10^9^pfu/100 μl control virus; and NS, equal volume of 0.9% NaCl administered in the same schedule as other groups. All the treatments were performed twice a week and lasted for 2 wk. Tumor width (W) and length (L) were measured every 4 d by calipers. The tumor volume (Tv) was calculated according to the following formula: Tv = 0.52 × L × W^2^. The treated mice were closely monitored and sacrificed if any signs of approaching death were shown. The mice in all groups were sacrificed 50 days after tumor establishment. All experiments involving mice were approved by the Institute's Animal Care and Use Committee.

### Detection of microvessel density and apoptosis

Frozen tissues were sectioned (5 μm) and fixed in acetone at 4°C. For detection of CD31 immunostaining, sections were probed with a monoclonal rat anti-mouse CD31 antibody (1:400, Santa Cruz Biotechnology, Santa Cruz, CA, US) at 4°C overnight, followed by incubation with biotinylated polyclonal goat anti-rat antibody (1:200, Vector Laboratories, Peterborough, UK) and Vectastain Elite ABC Kit (Vector Laboratories, Peterborough, UK). Positive reaction was visualized using 3,3-diaminobenzidine as chromagen (DAB substrate kit, Vector Laboratories, Peterborough, UK). Sections were counterstained with hematoxylin and mounted with glass coverslips. Apoptotic cells were identified using the TUNEL (terminal deoxynucleotidyl transferase-mediated nick end labeling) assay (In Situ Cell Death Detection Kit, Roche, Basel, Switzerland) following the manufacturer's guide. Images were captured by the Olympus fluorescence microscope at ×200 magnification. The quantification of microvessel density (MVD) (the maximum vascular area of the tumor) was assessed within hot spot[11]. The apoptotic cells were counted in 5 high power fields in each slide in a blinded manner. The percentage of apoptotic cells among tumor cells were calculated as apoptotic index.

### Alginate encapsulation assay

Alginate bead containing tumor cell assay was described in details previously[8]. Briefly, cultured LLC cells were resuspended with 1.5% (m/v) sodium alginate (Sigma-Aldrich, St. Louis, MO, US), and then the tumor cell alginate solution was dropped into a swirling bath of 0.25 M CaCl_2 _in order to form droplets containing about 1 × 10^5 ^tumor cells per bead. After anesthetized, the C57BL/6 mice were implanted s.c. with four beads into an incision on the back, the incisions were sutured with surgical clamps. Treatment of Ad-hEndo (1 × 10^9^pfu/100 μl) or cisplatin (1 mg/kg) was performed on day 0, 4, 8, 12 after bead implantation, with Ad-null or saline as control. At 14 days, the mice were injected i.v. with 100 μl FITC-dextran solution (Sigma Chemical) (100 mg/kg) and were sacrificed 20 minutes later. Image of the alginate implants was taken by using SPOT FIEX camera. Alginate beads were transferred to tubes containing 2 ml of saline. The tubes were mixed by a vortex for 20 s and centrifuged (3 min; 1000 × g). Finally the fluorescence of the supernatant was measured to quantify blood vessel formation.

### Toxicity Observation

Drug toxicity indexes such as weight loss, ruffled fur, behavior change and feeding patterns were continuously observed during the whole treatment.

To clarify potential side effects in the treated mice, the tissues of heart, liver, spleen, lung, kidney, etc., were fixed in 4% neutral buffered paraformaldehyde solution and embedded in paraffin. Sections of 3–5 μm were stained with hematoxylin and eosin (HE), and observed by two pathologists in a blinded manner.

As most adenoviruses infect liver tissues, we intratumorally injected viruses at 1 × 10^9 ^p.f.u./mouse, with cisplatin administration intraperitoneally. The operation schedule was the same as that for the animal experiments. After two-week treatment, blood samples were extracted from the tail vein. The white blood cell count, red blood cell count and platelet count were determined as measures of bone marrow toxicity, whereas creatinine, and GOT plus GPT were recorded as measures of kidney and liver toxicity, respectively.

### Statistical analysis

The results of the statistical analyses were presented as means ± standard deviation. For comparison of individual time points, differences between groups were tested by unpaired Student's t-test. Survival analysis was computed by the Kaplan-Meier method and compared by the log-rank test. All p values were two sides, and significant difference existed if p < 0.05.

## Results

### Expression of recombinant human endostatin in vitro

**Figure 1:**
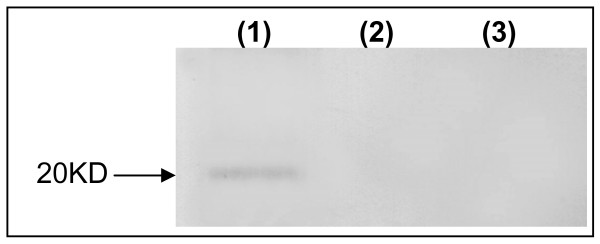
**Expression of recombinant human endostatin**. Recombinant human endostatin was expressed as a single band of appropriate 20 KD in Ad-hEndo transfected LLC cells(1), while no band was detected in Ad-null (2) transfected or untreated(3) tumor cells.

LLC cell line was transduced with 100 MOI of Ad-hEndo or Ad-null. 48 hr later, concentrated cultured supernatants were collected, mixed with 2× sample buffer, and then separated on a 12% SDS/PAGE gel. After transferred onto the PVDF membrane, followed by being incubated with the primary antibody and second antibody, a distinct band about 20 KD, corresponding to the volume of endostatin, was visualized in the Ad-hEndo treated cells, but not in Ad-null transduced and nontransduced cells (Figure 1).

### Combination treatment significantly reduced tumor growth and prolonged life span in vivo

**Figure 2:**
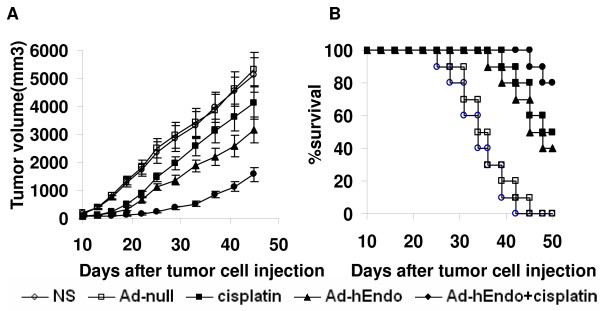
**Tumor suppression and survival advantage in C57BL/6 mice bearing LLC**. Tumor volume(A) and survival curves(B). Each group of Mice bearing LLC was s.c. injected intratumorally with corresponding treatment as described in "Methods". Treatment with combination of cisplatin and Ad-Endo resulted in the marked inhibition of tumor growth and longer life span(P < 0.05).

7 d after the Lewis lung cancer model was established, the C57BL/6 mice were randomized to receive administration with cisplatin, Ad-Endo, cisplatin plus Ad-Endo, Ad-null or NS (with the last two treatments as the controls). All mice were monitored every 4 d for changes in tumor growth. At Day 50, all the mice were sacrificed. Treatment with cisplatin or Ad-Endo as the single agent resulted in a 19.6% or 38.4% regression of tumor growth and prolonged survival time compared with the control groups (Ad-null or NS). Furthermore, the combination group showed reduced tumor volume by 69.5% and longer life span(P < 0.05) (Figure 2).

### Inhibition of tumor-induced angiogenesis and increase of apoptosis in vivo

**Figure 3:**
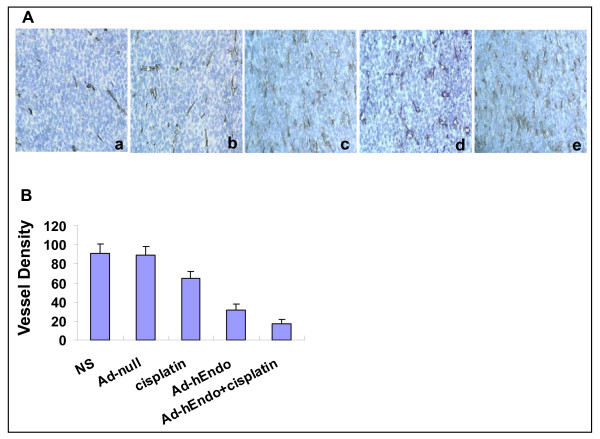
**Inhibition of angiogenesis within tumor estimated by CD31 immunohistochemical analysis**. (A) were representative sections from each group. a: Ad-hEndo+ cisplatin; b: Ad-hEndo; c: cisplatin; d: Ad-null; e: NS. (B) Vessel density was determined via counting the number of the microvessels per high-power field within hot spot area. Values were expressed as means ± SE (5 high power fields/slide). Tumors of the combination group showed smaller number of microvessel count than that of the other groups submitted to cisplatin or Ad-Endo alone, especially the NS (P < 0.05). a: Ad-hEndo+cisplatin; b: Ad-hEndo; c: cisplatin; d: Ad-null; e: NS.

**Figure 4:**
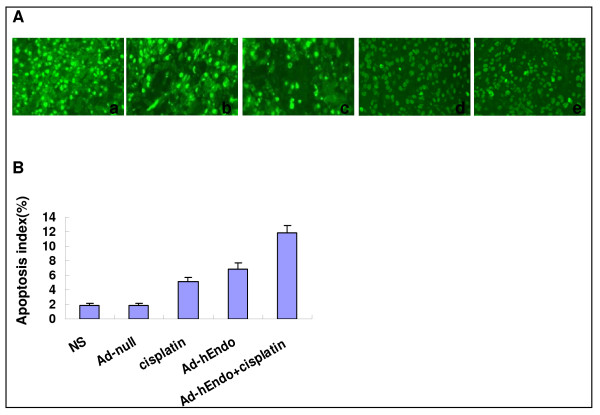
**Detection of apoptosis by terminal deoxynucleotidyl transferase-mediated dUTP nick-end labeling staining of tumor tissues**. (A) Sections after treatment were stained with the TUNEL analysis to detect apoptotic cells. (B) Apoptotic index was determined by calculating the percentage of apoptotic cells among tumor cells (5 high power fields/slide). The combination group showed the highest apoptotic index. a: Ad-hEndo+ cisplatin; b: Ad-hEndo; c: cisplatin; d: Ad-null; e: NS.

Angiogenesis within tumor tissues was estimated in terms of microvessel density (by counting the number of microvessels) on the section stained with anti-mouse CD31 antibody. The apoptotic tumor cells were determined by the TUNEL assay. Tumors of the control groups, treated with Ad-null or NS, showed larger microvessel count than those of the other groups submitted to cisplatin or/and Ad-Endo, especially the combination group (P < 0.05) (Figure 3). There was no difference in apoptotic index among all groups, but more apoptotic cells were seen in the group of chemotherapy or adenovirus treatment alone. Furthermore, the combination group showed the largest apoptotic index (Figure 4).

### Inhibition of angiogenesis in the alginate encapsulation assay

**Figure 5:**
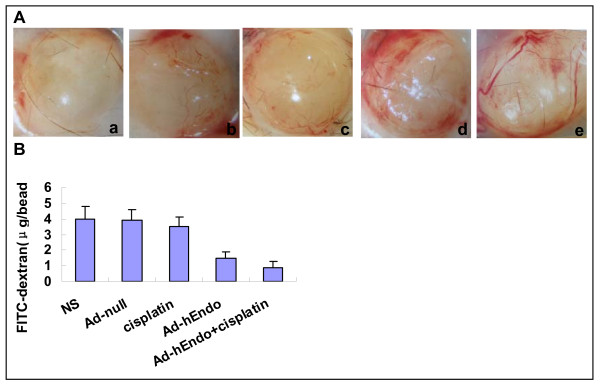
**Inhibition of antiangiogenesis assay by alginate bead in vivo**. (A) Representative alginate beads from each group. (B) FITC-dextran uptake of beads from each group. The combination group showed a significant decrease in vascularization compared with the control groups(P < 0.05). The results were expressed as mean ± S.E. a: Ad-hEndo+ cisplatin; b: Ad-hEndo; c: cisplatin; d: Ad-null; e: NS.

We examined the effect of endostatin on angiogenesis in vivo by the alginate encapsulation assay. Alginate beads containing lewis lung cancer cells were implanted s.c. on the back of C57BL/6 mice. Different treatments were performed in recipient mice. 14 d later, alginate implants containing LLC cells showed strong vascularization in the group of Ad-null or NS under the stereomicroscope. The FITC-dextran uptake was 62–77% higher in the group of Ad-null or NS than in the group of Ad-hEndo alone or in the combination treatment group and 11% more than in the group of cisplatin alone (Figure 5).

### Toxicity

**Figure 6:**
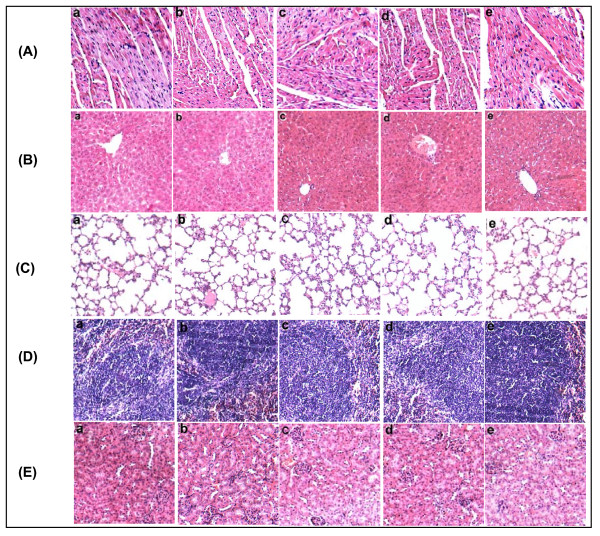
**Toxicity observation I**. H & E staining of heart(a), liver(b), lung (c), spleen(d) and kidney(e) in recipient mice. No hemorrhage in organs appeared in the combination group and no differences were seen among groups. A: Ad-hEndo+ cisplatin; B: Ad-hEndo; C: cisplatin; D: Ad-null; E: NS.

In the current research, compared with the control groups, no significant adverse consequences were observed in the light of gross measures such as weight loss, ruffled fur and behavior change. Furthermore, no pathologic changes in heart, liver, lung, spleen, kidney, etc., were found via microscopic examination(Figure 6).

The white blood cell count, red blood cell count and platelet count as well as GOT and GPT levels were all in the normal range. Compared with the control groups, none of the above parameters of the treatment groups showed significant difference (Table 1).

**Table 1:** Toxicity observation II

	**Ad-hEndo+ cisplatin**	**Ad-hEndo**	**cisplatin**	**Ad-null**	**NS**
White blood cell (×10^3^/mm^3^)	7.58 ± 2.12	7.89 ± 2.5	7.44 ± 1.98	7.96 ± 2.58	8.02 ± 2.83
Red blood cell (×10^4^/mm^3^)	701.5 ± 28.5	721.3 ± 22.5	700.4 ± 20.2	756 ± 25.2	780.5 ± 25.5
Platelet (×10^4^/mm3)	30.2 ± 7.5	25 ± 8.2	22.5 ± 6.9	32 ± 8.9	41 ± 7.2
					
GOT (IU/I)	241.3 ± 26.8	219.6 ± 35.6	252.6 ± 29.7	240.5 ± 39.4	267.5 ± 36.6
					
GPT (IU/I)	50.2 ± 11.3	43.2 ± 7.5	40.5 ± 7.9	42.8 ± 7.4	45.2 ± 8.4

Mean ± SD of 5 mice in each group. Compared with the control groups(p > 0.05), all treatment groups showed no significant difference.

## Discussion

It is well known that growth and progression of most solid tumors are angiogenesis dependent. Antiangiogenesis therapy for cancer can effectively inhibit tumor growth by inhibiting tumor-associated angiogenesis. When the tumor is deprived of essential nutrients and oxygen, the cell proliferation and metastasis is stalled. Endostatin is one of the potent endogenous angiogenesis inhibitors. Accumulated evidence suggests that it is a powerful specific marker of angiogenesis in malignancy of solid tumor. Although angiogenenic inhibitors can retard tumor growth through inhibiting angiogenesis, no angiogenesis associated agent alone or combination with other antitumor agents can eradicate tumor and reach desirable antitumor effects.

Chemotherapeutic agents exert damages to DNA and disrupt DNA replication in cell proliferation. Cisplatin, or cis-diamminedichloroplatinum (CDDP), as the adduct of platinum, has been an antineoplastic agent in general use. It shows similar mechanism as alkylation agent. The platinum-DNA crosslink kills cells in different cell cycles, inhibits DNA biosynthesis, and suppresses cell division after chloric ion is disassociated from the complex. Until now cytotoxic agents are the first-line drugs designated to kill the maximum tumor cells. However, chemotherapy in megadose is followed by serious side effects such as nausea, vomiting, hair loss, neurotoxicity and myelosuppression. In general, the responses in patients are unabiding with relapses accompanied by acquired resistance to the cytotoxic drugs in some heterogeneous survival cells because of indirect selection of chemotherapeutic drugs. At present the conventional dosing schedule is applied to balance the toxicity and efficacy, but the severe side effects and the ultimate failures remain refractory obstacles to administration of most chemotherapies. So new approaches are required to achieve a high therapeutic response rate.

A conventional dosing chemotherapy calls for episodic application of a cytotoxic drug, and requires a period of rest during chemotherapy to let normal cells recover. With a low rate of replication and cell division (the proliferation index of endothelial cells in tumor vessels is usually less than 3%), the tumor-associated endothelial cells are only weakly damaged in the standard chemotherapy. Tumor-related angiogenesis can supply essential nutrients and oxygen for the remaining tumor cells, which makes tumor relapse possible. Our current research confirmed that intratumoral injection of recombinant endostatin adenovirus plus a low dose of cisplatin could evidently improve antitumor efficacy, including tumor growth suppression, mice survival prolongation, and tumor cell apoptosis augmentation as well as neovascularization inhibition as compared with the controls. No serious adverse effects, such as ruffled fur, cachexia, anorexia, behavior change or toxic death were found in the combination group. However, up to now, the exact mechanism is not clear that how the combined agents induced anti-tumor efficacy. Two possible mechanisms may get involved. The first is induction of apoptosis. The antiangiogenic agents decrease supply of oxygen and nutrients for the tumor cells by reducing tumor vascular density, perfusion and vascular permeability[12], which leads to apoptosis of tumor cells and thus reinforces apoptosis efficacy of cisplatin. However, it is not clear whether the function of cisplatin in tumors is independent on gene transfer or is a specific part of adenovirus gene transfer. The second is antiangiogenesis. Cisplatin has been reported to influence the process of vascularization and to cause severe vasculotoxicity[13], which can strengthen the antiangiogenesis efficacy of endostatin. Low-dose cytotoxic treatment and antiangiogenesis therapy interact on each other. If the endothelial cells are treated by antiangiogenesis agents, they will lack certain adhesive contacts with matrix. Nonadherent endothelial cells are more susceptible to a cytotoxic agent, resulting in a higher apoptosis rate[14]. Meanwhile, the initial impaired angiogenesis can hamper and delay repair of inflicted dividing cells, and the increased vessel permeability may lead to increased tumor exposure to cytotoxic drugs. So tumor cells are more vulnerable to the damage effects of chemotherapy, especially when the cytotoxic drug is administered at a low dose[15,16]. Therefore, a coordination approach targeting multiple tumor-associated cell properties seems to be a promising strategy for marked inhibition of tumor growth[15,17-19].

In summary, our results in the current research indicate that the combination of antiangiogenesis gene therapy with low-dose chemotherapy was more effective to suppress tumor growth without obvious toxicity in mice than either agent alone. The mechanism may in part concern the increased induction of apoptosis and suppression of angiogenesis in the combination treatment. To our knowledge, it is the first time that the combination therapy of recombinant human endostatin adenovirus with low-dose cisplatin is administered and is found to have improved inhibitory effects on LLC mice. Therefore, the current study may lead to further exploration of potential application of combination strategy in lung cancer therapy. However, the optimum antiangiogenic agent and chemotherapeutic therapy dose to apply as well as the application schedule may remain unresolved [20-22]. Further researches are anticipated to choose the superior therapeutic combination strategy for lung cancer.

## Competing interests

The authors declare that they have no competing interests.

## Authors' contributions

RZB performed designed the project, cell culture, animal experiments and histologic analysis and drafted the manuscript. YW and QL prepared the recombinant human endostatin adenovirus and assisted with animal experiments. KX also contributed to animal experiments. YQW supervised experimental work and revised the manuscript. LY, YSW and KL helped to construct and produce the recombinant adenovirus. YL and JMS assisted with histologic analysis. BH participated in research design. JYL performed the statistical analysis. QL helped to draft the manuscript. NT and ZWZ carried out cell culture. All authors read and approved the final manuscript.
